# Genotypic and Antimicrobial Susceptibility of Carbapenem-resistant *Acinetobacter baumannii*: Analysis of IS*Aba* Elements and *bla*_OXA-23-like_ Genes Including a New Variant

**DOI:** 10.3389/fmicb.2015.01249

**Published:** 2015-11-13

**Authors:** Abbas Bahador, Reza Raoofian, Babak Pourakbari, Mohammad Taheri, Zahra Hashemizadeh, Farhad B. Hashemi

**Affiliations:** ^1^Department of Microbiology, School of Medicine, Tehran University of Medical SciencesTehran, Iran; ^2^Legal Medicine Research Center, Legal Medicine OrganizationTehran, Iran; ^3^Innovative Medical Research Center, Islamic Azad UniversityMashhad, Iran; ^4^Pediatrics Infectious Disease Research Center, School of Medicine, Tehran University of Medical SciencesTehran, Iran; ^5^Department of Microbiology and Virology, Shiraz University of Medical SciencesShiraz, Iran

**Keywords:** *Acinetobacter baumannii*, *bla*_OXA-23-like_ gene, carbapenemase, novel mutations

## Abstract

Carbapenem-resistant *Acinetobacter baumannii* (CR-AB) causes serious nosocomial infections, especially in ICU wards of hospitals, worldwide. Expression of *bla*_OXA_ genes is the chief mechanism of conferring carbapenem resistance among CR-AB. Although some *bla*_OXA_ genes have been studied among CR-AB isolates from Iran, their *bla*_OXA-23-like_ genes have not been investigated. We used a multiplex-PCR to detect Ambler class A, B, and D carbapenemases of 85 isolates, and determined that 34 harbored *bla*_OXA-23-like_ genes. Amplified fragment length polymorphism (AFLP) genotyping, followed by DNA sequencing of *bla*_OXA-23-like_ amplicons of CR-AB from each AFLP group was used to characterize their *bla*_OXA-23-like_ genes. We also assessed the antimicrobial susceptibility pattern of CR-AB isolates, and tested whether they harbored insertion sequences IS*Aba*1 and IS*Aba*4. Sequence comparison with reference strain *A. baumannii* (NCTC12156) revealed five types of mutations in *bla*_OXA-23-like_ genes; including one novel variant and four mutants that were already reported from China and the USA. All of the *bla*_OXA-23-like_ genes mutations were associated with increased minimum inhibitory concentrations (MICs) against imipenem. IS*Aba*1 and IS*Aba*4 sequences were detected upstream of *bla*_OXA-23_ genes in 19 and 7% of isolates, respectively. The isolation of CR-AB with new *bla*_OXA-23_ mutations including some that have been reported from the USA and China highlights CR-AB pervasive distribution, which underscores the importance of concerted national and global efforts to control the spread of CR-AB isolates worldwide.

## Introduction

Carbapenem-resistant *Acinetobacter baumannii* (CR-AB) can cause severe nosocomial infections particularly among patients in intensive care units (ICUs) around the world (Safari et al., 2013). Inadequate antimicrobial management of CR-AB infections often gives rise to highly resistant strains leading to prolonged hospitalization, treatment failures, and increased mortality (Higgins et al., 2010a). Epidemics of multi-, extensively-, and pandrug-resistant (MDR, XDR, and PDR) CR-AB have been reported from several countries (Kempf and Rolain, 2011; Bahador et al., 2013a; Moradi et al., 2015). In developing countries, such as Iran, challenges in the treatment of CR-AB infections are often exacerbated by widespread nosocomial outbreaks of OXA-type β-lactamase producing MDR-AB (for review see, Moradi et al., 2015). CR-AB are usually resistant to several β-lactams through the expression of chromosomal and plasmid-encoded carbapenemases including Ambler class A (*bla*_GES_, and *bla*_KPC_), class B (*bla*_IMP_, *bla*_NDM−1_, *bla*_SPM-1_, and *bla*_VIM_), and class D (*bla*_OXA-23, 40_, _and 58-like_; Siroy et al., 2005; Lu et al., 2009; Abbott et al., 2013). While the production of OXA-23 by *A. baumannii* is sufficient to confer resistance to carbapenems, insertion sequence (IS) elements IS*Aba*1 and/or IS*Aba*4 upstream of *bla*_OXA-23-like_ genes enhance the *bla*_OXA_–mediated carbapenem resistance of CR-AB (Turton et al., 2006; Lee et al., 2012; Evans and Amyes, 2014). Although there are a few reports from Iran regarding the distribution and/or frequency of the *bla*_OXA-51-like_ genes among CR-AB, data about characterization of their *bla*_OXA-23_ genes and IS*Aba* elements is not available.

In this study, we have characterized *bla*_OXA_ genes in CR-AB isolates from Iran, and report new variants that harbor novel mutations in their *bla*_OXA-23-like_ carbapenemase genes. In addition to analyzing the distribution and frequency of *bla*_OXA-23-like_ genes, we have determined the antimicrobial susceptibility patterns of isolates and the presence of IS*Aba*1 and IS*Aba*4 enhancer elements upstream of their *bla*_OXA-23-like_ genes. Characterization of *bla*_OXA_ genes and assessment of carbapenemase-mediated antibiotic resistance among *A. baumannii* isolates can help efforts to develop databases, which are essential to a comprehensive national surveillance program in Iran, toward the local and global control of CR-AB outbreaks.

## Materials and methods

### Specimens and bacterial isolates and cultures

A total of 85 non-repetitive clinical specimens were collected during 2011 from the intensive care units (ICUs) of Imam Khomeini Medical Center (IKMC) and Children's Medical Center (CMC) in Tehran, Iran. IKMC and CMC are affiliated with Tehran University of Medical Sciences (TUMS), and both are large referral centers that provide tertiary health care to patients from all over Iran. Specimens were collected from ICUs in surgical (S), internal medicine (M), emergency (E), pediatrics (P), and kidney transplantation (T) wards. Clinical isolates were initially identified as *A. baumannii* using the API20NE system (bioMérieux, Marcy-l'Etoile, France), and were further confirmed by *gyrB* multiplex PCR, as described previously (Higgins et al., 2010b). Specimen sources for *A. baumannii* isolates were as follows: respiratory tract (*n* = 51), urine (*n* = 16), blood (*n* = 11), wound (*n* = 5), and cerebral spinal fluid (CSF; *n* = 2). Twenty six of the *A. baumannii* isolates were part of a previous molecular epidemiologic study (Bahador et al., 2014). Brain heart infusion (BHI) agar plates and Mueller-Hinton broth (MHB; both from Merck, Germany) were used to culture the bacterial isolates.

### Antimicrobial susceptibility testing

To assess susceptibility of *A. baumannii* clinical isolates, the disk agar diffusion (DAD) method (CLSI, 2015) was carried out according to the Clinical and Laboratory Standards Institute (CLSI) procedures and breakpoint interpretations, using antimicrobial disks containing 19 different antimicrobial agents (Mast Diagnostics, Bootle, UK; **Table 2**). The CLSI guideline for broth microdilution test for minimum inhibitory concentrations (MICs) was used to assess the susceptibility of MDR-AB isolates to colistin (CST), imipenem (IPM), rifampicin (RIF), and tigecycline (TGC). For tigecycline susceptibility tests, the criteria of the European Committee on Antimicrobial Susceptibility Testing (EUCAST) for *Enterobacteriaceae* were used, in which an MIC of < 1 μg/mL was defined as susceptible and >2 μg/mL was considered resistant (EUCAST, 2015). Rifampicin susceptibility was interpreted according to CLSI criteria using breakpoint values suggested for *Staphylococcus aureus*, in which susceptible and resistant were defined as ≤ 1 μg/mL and ≥4 μg/mL, respectively (CLSI, 2015). *A. baumannii* isolates were defined as MDR, XDR, and PDR according to the definitions provided by Magiorakos (Magiorakos et al., 2012). The MIC geometric mean (MIC_gm_) of imipenem against *bla*_OXA-23-like^+^_ CR-AB isolates were also compared with the MIC_gm_ of non-mutant isolates, and fold-increase calculations were measured against MIC_gm_ of non-mutant strains.

### Detection of carbapenemase gene IS*Aba*1 and IS*Aba*4 insertion sequences

The overall strategy for the identification of the 34 *bla*_OXA-23-like^+^_ CR-AB isolates is shown in Supplemental Figure 1. Briefly, we tested all 85 *A. baumannii* isolates for carbapenemase production by the modified Hodge test (Lee et al., 2012), and their chromosomal DNA were tested by two different confirmatory multiplex-PCR assays to identify the most common carbapenemase encoding genes. The criteria to include isolates in this study were the presence of PCR-specific amplicons, confirmed by agarose gel electrophoresis analysis (Supplemental Figure 1). A novel in-house multiplex-PCR, referred to as AB-hexaplex-PCR was optimized for the rapid and simultaneous detection of the most common carbapenemase genes, including Ambler class A and B (*bla*_KPC_, *bla*_GES_, *bla*_IMP-1_, *bla*_VIM-2_, *bla*_NDM-1_, and *bla*_SPM-1_) in *A. baumannii* using Primer 3 software (version 4.0; http://primer3.wi.mit.edu/; accessed June 05, 2011). Reference gene sequences were accessed from GenBank [http://www.ncbi.nlm.nih.gov/GenBank (*bla*_KPC_: GQ140348, *bla*_GES_: GU207844, *bla*_IMP-1_: EF375699, *bla*_VIM-2_: GQ288396, *bla*_NDM-1_: JN794561, and *bla*_SPM-1_: HM370523; accessed June 04, 2011)], as shown in Table 1. The Ambler class D type carbapenemase genes (*bla*_OXA-23, 24, 51, 58 like_) were detected using the Woodford multiplex PCR assay method (Woodford et al., 2006). Additionally, the AB-hexaplex-PCR distinguished amplicons corresponding to the *bla*_IMP-1_, *bla*_SPM-1_, *bla*_GES_ and *bla*_KPC_, *bla*_NDM-1_, and *bla*_VIM-2_ genes, and isolates that harbored these genes were excluded from our study (representative gel; Supplemental Figure 2). The frequency of IS*Aba*1 and IS*Aba*4 elements upstream of *bla*_OXA-23-like_ and *bla*_*OXA*-51-like_ genes were assessed using a series of PCR amplifications. A set of primers, referred to as IS*Aba*1F/OXA-23R, IS*Aba*4F/OXA-23R, and IS*Aba*1F/OXA-51R, is shown in Table 1. After our serial screening of isolates, 34 isolates were identified that harbored *bla*_OXA-23-like_ gene as their sole acquired carbapenemase gene.

**Table 1:** **Primer sequences and adaptors (and their corresponding reference) utilized in order to identify most common carbapenemase genes in our isolates, and to generate amplicons for AFLP genotyping analysis**.

**Assay**		**Primer**	**Sequence (5′-3′)^†^**	**Size of amplicon**	**References**
Detection of carbapenemase in the molecular class D	Multiplex PCR	*bla*_*OXA*-51_likeF	TAATGCTTTGATCGGCCTTG	353	Woodford et al., 2006
		*bla*_*OXA*-51_likeR	TGGATTGCACTTCATCTTGG		
		*bla*_*OXA*-23_likeF	GATCGGATTGGAGAACCAGA	501	"
		*bla*_*OXA*-23_likeR	ATTTCTGACCGCATTTCCAT		
		*bla*_*OXA*-24_likeF	GGTTAGTTGGCCCCCTTAAA	240	"
		*bla*_*OXA*-24_likeR	AGTTGAGCGAAAAGGGGATT		
		*bla*_*OXA*-58_likeF	AAGTATTGGGGCTTGTGCTG	590	"
		*bla*_*OXA*-58_likeR	CCCCTCTGCGCTCTACATAC		
Detection of carbapenemase in the molecular classes A and B	hexaplex PCR (h-PCR)	IMP-1F	AACATGGTTTGGTGGTTCTTGT	263	Present study
		IMP-1R	TCCGCTAAATGAATTTGTGGCT		
		VIM-2F	CAATGGTCTCATTGTCCGTGAT	395	"
		VIM-2R	AAATCGCACAACCACCATAGAG		
		NDM-1F	CTGGATCAAGCAGGAGATCAAC	118	"
		NDM-1R	ATTGGCATAAGTCGCAATCCC		
		KPCF	CGCTAAACTCGAACAGGACTTT	640	"
		KPCR	ATAGTCATTTGCCGTGCCATAC		
		blaGESF	GAAAACTTTCATATGGGCCGGA	567	"
		blaGESR	GACCGACAGAGGCAACTAATTC		
		SPM-1F	CCATTGTCTGCAAAAAGTTCGG	439	"
		SPM-1R	AAACATTATCCGCTGGAACAGG		
*ISAba1 detection upstream of blaOXA-51*	*IsAba-1 F/OXA-51 R*	IsAba-1 F	AAGCATGATGAGCGCAAAG	227	"
		OXA-51 R	GGTGAGCAGGCTGAAATAAAA		
ISAba1 detection upstream of *bla*OXA-23	IsAba-1 F/OXA-23 R	IsAba-1 F	TGAGATGTGTCATAGTATTC	314	"
		OXA-23 R	AGAGCATTACCATATAGATT		
*ISAba4* detection upstream of *bla*OXA-23	IsAba-4 F/OXA-23 R	IsAba-4 F	CACAATTTCTGATAAAGATA	327	"
		OXA-23 R	TTTATTAAATTATGCTGAAC		
AFLP	Adaptors	adp MbI	GTAGCGCGACGGCCAGTCGCG	No amplicon	Bahador et al., 2013b
		ADP MbI	GATCCGCGACTGGCCGTCGCGCTAC		
		adp MsI	GTAGCGCGACGGCCAGTCGCGT		"
		ADP MsI	TAACGCGACTGGCCGTCGCGCTAC		
	Pre-amplification	PreAmp Mbo	ACGGCCAGTCGCGGATC	Multiple and variable	"
		PreAmp Mse	CGACGGCCAGTCGCGTTAA		
	Selective primers	Mbo1	PreAmp Mbo + A	Multiple and variable	"
		Mbo2	PreAmp Mbo + T		
		Mbo3	PreAmp Mbo + C		
		Mbo4	PreAmp Mbo + G		
		Mse1	PreAmp Mse + A		
		Mse2	PreAmp Mse + T		
		Mse3	PreAmp Mse + C		
		Mse4	PreAmp Mse + G		

† *Nucleotide*.

### AFLP genomic fingerprint analysis

Amplified fragment length polymorphism (AFLP) genotyping of *bla*_OXA-23-like^+^_ and *bla*_OXA-51-*like*^+^_ isolates was carried out by a modified Vos method (Vos et al., 1995), as described previously (Bahador et al., 2013b). AFLP typing was carried out prior to sequence analysis to ensure thorough examination of the diversity of CR-AB isolates. Briefly, chromosomal DNA was size-verified and double-digested with MboI and MseI (Fermentas, Lithuania). Then DNA fragments were ligated to corresponding adapters using T4 DNA ligase (350 U/μL, Takara Bio, Japan) followed by the preliminary PCR using PreAmp-Mbo and PreAmp-Mse primers (Table 1). Preliminary PCR amplicons served as templates for selective PCR, which generated AFLP genotype profiles upon agarose gel analysis. Initial testing of 36 combinations of primers, including PreAmp Mbo (PreAmp Mbo+A, +T, +C, +G), and PreAmp Mse (PreAmp Mse +A, +T, +C, +G) and *A. baumannii* NCTC12156 DNA as a normalization reference showed that the Mbo4-Mse4 combination generated the clearest AFLP profiles when analyzed using BioNumerics version 5.10 (Applied Maths, Sint-Martens-Latem, Belgium). The similarity between band patterns was calculated using the Dice coefficient, with an optimization of 0.5% and a position tolerance of 1%. The AFLP types were grouped at the 90% similarity cutoff on a dendrogram constructed by the unweighted-pair group method using average linkages (UPGMA).

### DNA sequencing of *bla*_OXA-23-like_ genes

To evaluate an association between changes in the chromosomal carbapenemase gene sequence of isolates and their antimicrobial resistance pattern, a two-step approach was adopted. An initial AFLP assay was carried out on *bla*_OXA-23-like^+^_ CR-AB, followed by DNA sequence analysis of the *bla*_OXA-23-like_ gene of a representative isolate from each AFLP genotype group.

Briefly, we used a high fidelity Pfu DNA polymerase (Fermentas, Lithuania) to generate *bla*_OXA-23-like_ specific amplicons, which were purified using a AccuPrep® PCR Purification Kit (Bioneer, Daejeon, Korea) and cloned into pTZ57R (InsT/A Clone PCR product cloning kit, Fermentas, Vilnius, Lithuania). DNA was then transferred into competent *E. coli* TOP10 cells, which were then isolated using Luria-Bertani (LB) agar supplemented with ampicillin (100 μg/mL). Plasmid DNA was prepared with the AccuPrep Plasmid MiniPrep DNA Extraction Kit, (Bioneer, Daejeon, Korea) and sequenced using an ABI3730 automatic sequencer (Applied Biosystems, CA, USA). The sequences were analyzed using a BLAST algorithm against the NCBI GenBank database [http://www.ncbi.nlm.nih.gov/guide/dna-rna/ (accessed 05.06.11)].

### Iodometric assay of β-lactamase activity

Bacterial β-lactamase enzymatic activity was determined by an iodometric assay, as described previously (Sawai et al., 1978). Briefly, crude lysates of 16 isolates that represented AFLP groups were extracted using the Saino method (Saino et al., 1982). Briefly, overnight bacterial growth were diluted in MHB broth to a concentration of 10^7^ cfu/ml and incubated in a shaker for 2 h at 35°C. As an inducer, imipenem was added at 0.25 of the isolate MIC and incubated for an additional 2 h (Clark, 1996). Bacterial cells were harvested, centrifuged at 4°C, washed twice with 50 mM phosphate buffer saline (PBS; pH 7.0), and re-suspended in 0.1 M PBS (pH 7.0). The suspension was sonicated in an ultrasonic disrupter (Branson Ultrasonics Co., Shanghai, China) at 75 W for 3 min in an ice bath; afterward the disrupted cell suspension was centrifuged at 13,000 × g for 30 min at 4°C. The β-lactamase enzymatic activity of the supernatant fluid (i.e., bacterial lysate) was measured against imipenem using iodometric method described by Doust (Daoust et al., 1973) using reagents prepared, as described previously (Onishi et al., 1974; Sawai et al., 1978; Minami et al., 1980). Briefly, iodine reagent (40 μmol in 0.5 M acetate buffer, pH 4.0) was added to lysate supernatant fluids, after 5 min incubation with imipenem (50 μg/mL) at 30°C. Ten minutes later, samples' absorbance was measured at 620 nm, and imipenem hydrolysis was determined. Activity was reported as the mean of triplicate samples in micromoles of imipenem degraded per minute per milligram of protein in each bacterial extract. Protein concentrations were measured by Bradford assay kit (Pierce™ Coomassie Plus Assay Kit, Thermo Scientific, Ottawa, Canada).

### *In silico* analysis and nucleotide sequence accession numbers

*In silico* analysis was carried out using GenBank nucleotide database. Predict Protein software (hosted by Rostlab) was also used to predict changes as a result of a frameshift mutation. The nucleotide sequence data were deposited in the GenBank nucleotide database under accession numbers: JQ343842.1, JQ343840.1, JQ343838.1, JQ343836.1, JQ343841.1, JQ343839.1, JQ343837.1, JQ360584.1, JQ360582.1, JQ360580.1, JQ360578.1, JQ360583.1, JQ360581.1, JQ360579.1, JQ360577.1, and JQ061320.1. The novel DNA sequence of *bla*_OXA 23_ genes, with “No Full-Match” by GenBank; as well as its corresponding peptide amino acid sequence was submitted to Lahey database (lahey.org/Studies).

## Results

### Antimicrobial susceptibility profiles and AFLP genomic fingerprint analysis

Table 2 shows the susceptibility profiles of all 85 CR-AB isolates against CLSI groups of antimicrobial agents. Overall, CR-AB isolates were most resistant to CLSI group A (51–96%), followed by group B (25–97%) antimicrobials. Overall, up to 96% of isolates were resistant to 12 of the tested antimicrobials; while the rates of resistance to tigecycline, imipenem, and doripenem were 34, 65, and 94%, respectively. The lowest resistance rates among isolates were against colistin (12%), minocycline (25%), and doxycycline (31%). Interestingly, all colistin-resistant isolates were susceptible to tigecycline and/or tobramycin. The frequency of MDR, XDR and PDR isolates were 69, 24, and 0%, respectively; and broadly-resistant CR-AB isolates were most frequently recovered from the surgical and internal medicine ICU wards. However, the frequency of resistant isolates was generally proportional to the number of specimens from each ICU (Table 2).

**Table 2:** ***In vitro* antimicrobial susceptibility results of 85 non-replicate clinical *A. baumannii* isolates according to CLSI antimicrobial grouping of A, B, and O, as according to the frequency of *A. baumannii* isolation from the type of ICU ward**.

**ICU Ward (No.)**	**% Resistant to CLSI antimicrobial groups^a^**																						
	**A**									**B**											**O**		
	**IPM**^b^	**DOR**	**SAM**	**CRO**	**CAZ**	**TOB**	**GEN**	**CIP**	**AMK**	**MIN**	**DOX**	**TET**	**TGC**	**LVX**	**PIP**	**CTX**	**FEP**	**TZP**	**TIM**	**SXT**	**CST**	**NET**	**RIF**
Emergency (12)	8	13	9	14	14	7	12	11	12	5	5	9	8	6	14	14	13	11	14	14	1	6	13
Medical Care (27)	25	30	20	31	32	22	27	22	27	7	7	16	5	19	27	27	33	27	33	32	4	12	27
Pediatrics (8)	6	9	5	9	9	5	9	6	5	1	1	6	4	6	8	7	9	9	8	9	1	5	8
Surgical (31)	22	34	20	34	34	13	29	28	31	11	14	21	13	19	33	33	35	29	33	33	5	14	33
Transplantation (7)	4	8	5	8	7	4	6	7	7	1	4	5	5	5	7	8	7	4	8	8	1	2	7
Total (85)	65	94	59	96	96	51	82	74	81	25	31	58	34	56	89	89	97	80	96	96	12	39	88

a *Criteria in the assignment of agents to Groups A, B, and C included clinical efficacy, prevalence of resistance, minimizing emergence of resistance, cost, FDA clinical indications for usage, and current consensus recommendations for first-choice and alternative drugs. **Group A** are considered appropriate for inclusion in a routine, primary testing panel, as well as for routine reporting of results for the specific organism.**Group B** comprises agents that may warrant primary testing. **Group O (Other)** includes agents that have a clinical indication for the organism. Escherichia coli ATCC25922 and Pseudomonas aeruginosa ATCC27853 were used for quality control of antimicrobial susceptibility testing and included in each run*.

b *CSF, cerebrospinal fluid; AMK, amikacin; CAZ, ceftazidime; CIP, ciprofloxacin; CRO, ceftriaxone; CST, colistin; CTX, cefotaxime; DOR, doripenem; DOX, doxycycline; FEP, cefepime; GEN, gentamycin; IPM, imipenem; MIN, minocycline; NET, netilmicin; LVX, levofloxacin; PIP, piperacillin; RIF, rifampicin; SAM, ampicillin–sulbactam; SXT, trimethoprim/sulfamethoxazole; TET, tetracycline; TGC, tigecycline; TZP, Piperacillin/tazobactam; TIM, ticarcillin/clavulanic acid; TOB, tobramycin*.

**Figure 1:**
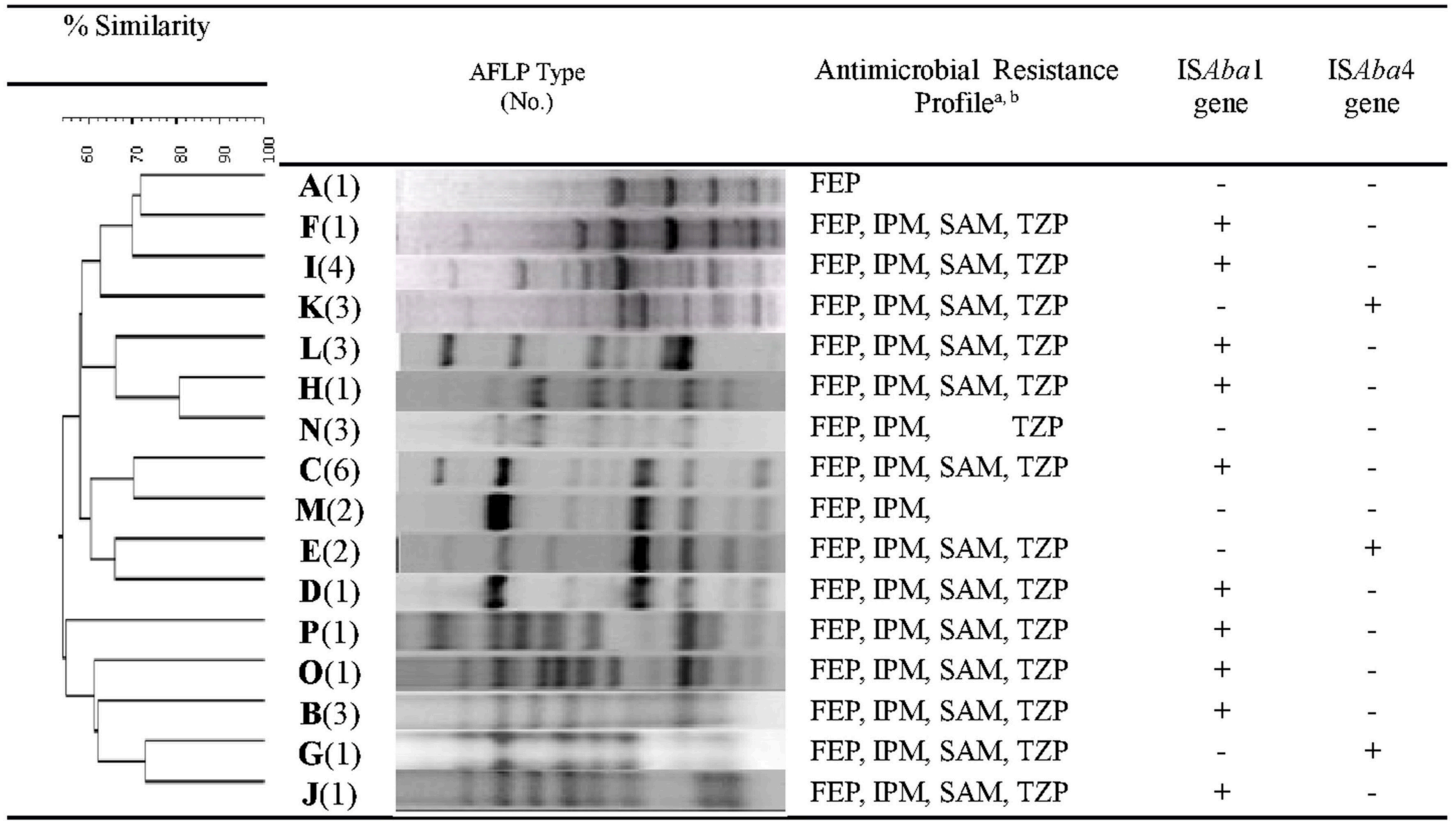
**Dendrogram of amplified fragment length polymorphism (AFLP) analysis of genomic DNA extracted from *bla*^(+)^_OXA-23-like_ CR-AB isolates**. Susceptibility to select antimicrobials and IS*Aba* presence is also indicated. Dice coefficient with 0.5% optimization and 1% position tolerance was used. Dendrogram was constructed by unweighted-pair group method using average linkages (UPGMA); and AFLP type identification was defined by groups formed at 90% similarity cutoff. ^*a*^FEP, cefepime; IPM, imipenem; SAM, ampicillin-sulbactam; TZP, Piperacillin/tazobactam. ^*b*^All 16 AFLP types were resistant to the following beta-lactam antimicrobial agents: CAZ, ceftazidime; CRO, ceftriaxone; CTX, cefotaxime; PIP, piperacillin; TIM, ticarcillin/clavulanic acid.

Our analysis revealed that 34 (40%) isolates harbored *bla*_OXA-23-like_ genes, and 51 isolates were resistant to carbapenems but did not harbor *bla*_OXA-23-like_ genes (Supplemental Figure 1). AFLP genotype analysis of *bla*_OXA-23-like^+^_ isolates generated 16 distinct AFLP genotypic groups, labeled genotype A through P. Group C (*n* = 6) was the predominant AFLP type, followed by genotype I (*n* = 4), and genotypes B, K, L, and N (*n* = 3 in each group). While each AFLP group consisted of 1 to 6 isolates, 50% (8/16) of the groups consisted of a single isolate, indicative of a high diversity among CR-AB isolates. Despite this diversity, the antimicrobial susceptibility patterns among 13 (82%) genotypes were similar, with the exception of genotypes A, M, and N (Figure 1).

### Detection of IS*Aba*1 and IS*Aba*4

Overall, IS*Aba*1 and IS*Aba*4 sequences were present in 61% and 7% of all tested CR-AB isolates, respectively. Among 34 *bla*_OXA-23-like^+^_ isolates, 67% were IS*Aba*1^+^, and 18% were IS*Aba*4^+^ (Figure 1); whereas 10 (63%) and 3 (19%) of AFLP genotypes harbored IS*Aba*1 and IS*Aba*4 elements, respectively. IS*Aba*4 was only present among AFLP types G, E, and K isolates, while genotypes A, M, and N has neither IS*Aba*1, nor IS*Aba*4 element (Table 3; Figure 1). Interestingly, even though the IS*Aba*1^+^ isolates of genotype B (*N* = 3) exhibited an XDR profile, all genotype B isolates remained susceptible to tobramycin and ampicillin–sulbactam.

**Table 3:** **Comparison of *bla*_OXA-23-like_ gene sequences among CR-AB isolates belonging to various AFLP genotype groups, as compared to *bla*_OXA-23_ gene sequence of *A. baumannii* reference strain, according to the specimen source, MIC against imipenem, β-lactamase activity, and IS*Aba* element status**.

**No**.	**Isolates**	**AFLP type (No.)**	**Specimen (ICU)^a^**	**Nucleotide Change(s)**	**Amino Acid Change(s)**	**MIC of IMP (mg/L)**	**Mean of β-lactamase activity^d^ (SD)^e^**	**IS^b^ type**	
								**IS*Aba* 1**	**IS*Aba* 4**
1	*bla*_OXA-23_	N (3)	Urine (S)	None	None	8	ND	−	−
2	"	M (2)	Urine (S)	"	"	16	1.05(0.10)	−	−
3	"	A (1)	Urine (S)	"	"	16	0.87(0.04)	−	−
4	"	H (1)	Blood (P)	"	"	32	2.63(0.15)	+	−
5	"	I (4)	Blood (E)	"	"	16	1.35(0.09)	+	−
6	"	B (3)	CSF (S)	"	"	16	ND	+	−
7	*bla*_*OXA*-482_	F (1)	Urine (M)	Insertion of A at position 335 and deletion of A at position 336	Ser 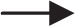 Tyr at aa 112	32	0.42(0.04)	+	−
8	"	G (1)	Urine (T)	"	"	16	5.58 (0.91)	−	+
9	"	O (1)	Urine (E)	"	"	16	2.11(0.63)	+	−
10	"	P (1)	Sputum (E)	"	"	16	2.35(0.87)	+	−
11	*bla*_*OXA*-481_	E (2)	Wound (S)	G 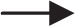 A at position 771	Met 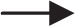 Ile at aa 257	64	1.67(0.09)	−	+
12	"	C (6)	Urine (S)	"	"	128	7.96(0.92)	+	−
13	"	K (3)	Blood (M)	"	"	64	7.75(1.03)	−	+
14	*bla*_*OXA*-495_	D (1)	Sputum (S)	Frame-shift due to insertion of A at position 335 and deletion at A at position 354	Change in aa112–aa118 motif^c^	128	14.37(1.33)	+	−
15	*bla*_*OXA*-366_	J (1)	Blood (M)	A 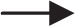 C at position 376, and G 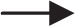 A at position 625	Met 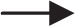 Ile at aa 126, and Glu 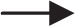 Lys at aa 209	16	2.62(0.53)	+	−
16	*bla*_*OXA*-422_	L (3)	Sputum (S)	G 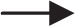 A at position 766	Glu 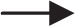 Lys at aa 256	16	0.37(0.03)	+	−

a *ICUs: E, Emergency; M, Medical Care; P, pediatric; S, surgical; T, transplantation*.

b *IS: Insertion sequence*.

c *SFTAWE
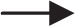
YIYRLG*.

d *μmole imipenem hydrolyzed per min per mg of protein*.

e *standard deviation*.

*ND, No detactable activity*.

Among the *bla*_OXA-23-like^+^_ isolates that harbored either IS*Aba*1 or IS*Aba*4 elements, a majority (82%) displayed a distinctive profile of resistance to 9 antimicrobial agents, namely, CAZ, CRO, CTX, DOR, FEP, IPM, PIP, SAM, and TZP (Figure 1). The presence of either IS*Aba*1, or IS*Aba*4 imparted resistance to imipenem and doripenem among *bla*_OXA-23-like^+^_ genotypes. However, the genotype A isolate (which was IS*Aba*1^−^ and IS*Aba*4^−^) also showed resistance to imipenem (MIC = 16μg/mL).

### Sequence analysis of *bla*_OXA-23-like_ genes

Table 3 demonstrates sequence differences between the *bla*_OXA-23-like_ specific amplicon among members of AFLP groups vs. the *bla*_OXA-23_ sequence of the *A. baumannii* reference strain (referred to as “wild type”). The *bla*_OXA-23_ gene sequences of six (37%) AFLP genotypes did not differ from the wild-type strain; however, isolates from 10 (63%) AFLP types had mutations in *bla*_OXA-23_ genes. Five different *bla*_OXA-23_ gene mutations were detected, and a Genbank search revealed that one of the mutant sequences had been recently reported from the USA, namely *bla*_OXA-366_, and three were reported from China (i.e., *bla*_OXA-422_, _481_, _and 482-like_ genes). One novel *bla*_OXA-23-like_ gene sequence was thus submitted to the Lahey database and assigned as the *bla*_OXA-495-like_ gene.

At the points of carbapenemase gene mutations, comparison of a 21-nucleotide sequence of PCR amplicons obtained from isolates in mutant AFLP groups that had >2 members, i.e., groups L, E, C, and K, showed strong homologies within these AFLP types (Supplemental Figure 3). Additionally, Table 3 displays the AFLP type, imipenem MIC, and IS*Aba* status among various *bla*_OXA-23^+^_ isolates according to their specimen source. The absence of mutation(s) in *bla*_OXA-23_ genes was associated with lower imipenem MICs as compared to mutant isolates. Imipenem MIC_gm_ for all non-mutant *bla*_OXA-23_ gene isolates was 16 μg/mL (range = 8−32μg/mL), whereas the mean MIC for mutants was almost 50 μg/mL (range = 16−128μg/mL). Conversely, alterations of the *bla*_OXA-23_ gene were associated with an increased MIC to imipenem among *A. baumannii* isolates. While 16 (47%) of *bla*_OXA-23-like^+^_ isolates harbored IS*Aba*1 sequences upstream of their carbapenemase gene, only six (18%) isolates were IS*Aba*4^+^. IS*Aba*1 elements were detected upstream of either *bla*_OXA-23_ or *bla*_*OXA*-51_ genes, orboth these genes. Since, none of the *bla*_OXA-23-like^+^_ isolates were both IS*Aba*1^+^ and IS*Aba*4^+^, the presence of these elements appeared mutually exclusive among isolates (Table 3). The IS*Aba*4 sequence was absent among *bla*_OXA-23-like_ genes of non-mutant CR-AB isolates, whereas six of mutant isolates, namely genotypes G, E, and K, were IS*Aba*4^+^ with moderate MICs, ranging from 16 to 64 μg/mL (Table 3). Although mutant isolates of genotypes C and D were IS*Aba*1^+^ had the highest imipenem MICs (128 μg/mL), the imipenem MIC of IS*Aba*1^+^ mutants among genotypes O, P, J, and L was 16 μg/mL. Furthermore, all IS*Aba*1^−^ and IS*Aba*4^−^ CR-AB isolates had non-mutant *bla*_OXA-23^+^_ genes, namely genotypes A, M, and N isolates, which showed the lowest imipenem MICs (8–16 μg/mL), as well. Surprisingly, three *bla*_OXA-23-like^+^_ isolates (genotypes A and M) had imipenem MICs of 16 μg/mL, but harbored neither IS*Aba*1, nor IS*Aba*4 elements upstream of the *bla*_OXA-23-like_ gene, suggestive of other resistance mechanisms in these isolates (Table 3).

As shown in Table 3, among mutations of the *bla*_OXA-23-like_ genes, insertions/deletions at nucleotide positions 335 and 336 were most frequent (40%), followed by a single substitution at position 771 (30%). Isolates with F, G, O, and P genotypes showed the insertion/deletion mutations at position 335, with imipenem MICs of 16–32 μg/mL, whereas isolates with genotypes E, C, and K had the substitution at position 771 and showed the highest imipenem MICs of 64–128 μg/mL (Table 3). Further analysis revealed that three other single substitutions also occurred at positions 376, 625, and 766 among genotypes J and L, but their imipenem MICs were not higher than non-mutants (16 μg/mL). In addition, three isolates with the substitution at position 771 had a 3–4-fold increase in imipenem MIC over that of the non-mutants. A frame-shift mutation at position 355, which corresponds to a change at aa112–118, in genotype D isolate (strain TUMS/BTRF 661) was associated with a four-fold increase in imipenem MIC_gm_ (128 μg/mL) over that of non-mutant isolates. Software prediction showed that the frame-shift mutation may change the subcellular localization of carbapenemase and enhance its secretion rate, which can explain the high MIC of the isolate against carbapenems. *In silico* comparison of carbapenemase binding domains of wild-type vs. the frame-shift mutant also showed that the binding domain changed from “aa15–24 and aa28–29” to “aa15–22 and aa24–25” motif, which may lead to higher affinity of mutant OXA-23 enzyme for the binding cleft of carbapenems.

Five *bla*_OXA-23_ mutants and one non-mutant isolate showed imipenem MICs of >16 μg/mL. Further *in silico* analysis revealed that the *bla*_OXA-23-like_ gene mutations would lead to up to six amino acid changes in the carbapenemase protein. However, the highest imipenem MICs were associated with a single substitution at position 771 of *bla*_OXA-23-like_ gene, corresponding to aa 257 substitution in carbapenemase among genotypes E, C, and K, which represented 55% of the mutant isolates. All AFLP types with non-mutant *bla*_OXA-23_ genes showed MICs of ≤ 16 μg/mL for imipenem, except the genotype H isolate (MIC = 32μg/mL); however, all *bla*_OXA-23-like_ mutants showed high imipenem resistance (MIC = 16–128 μg/mL).

As shown in Table 3, we compared the β-lactamase activity of the 16 representative isolates from each AFLP group. Overall, the range of β-lactamase activity of non-mutant isolates was lower (not detectable–2.63 μmoles/min/ mg protein) than the *bla*_OXA-23-like_ mutants (0.37–14.37 μmoles/min/mg protein). Mutant isolates, such as the frame-shift mutant genotype D, showed the highest imipenem MICs (i.e., 64 and 128 μg/mL), as well as the highest β-lactamase activities, i.e., 7.96 and 14.37 μmoles/min/mg protein for genotype C and K, respectively. In contrast, lysates from isolates with no *bla*_OXA-23-like_ gene mutation that had the lowest MICs (e.g., genotypes N and B), and showed no detectable β-lactamase activity. The β-lactamase activity of a imipenem-susceptible (MIC < 4 μg/ml), clinical *A. baumannii* isolate, was also below assay's detection level (data not shown). Among the 4 isolates that had the same mutation at position 335, the β-lactamase activity was between 0.42 and 5.58 μmoles/min/ mg protein, while imipenem their MIC was 16–32 μg/mL. However, with the exception of genotype E, the β-lactamase activity of mutants with position 771 mutation, was increased concomitant with high MICs in these isolates. The majority (70%) of mutant isolates were recovered from two ICU wards; namely, the surgical (*n* = 4), and the internal medicine (*n* = 3) ICU; however, no mutant isolates were recovered from the pediatric ICU ward. Among the *bla*_OXA-23-like_ mutant isolates, eight (80%) were recovered from either urine or sputum specimens (Table 3).

Table 4 shows the distribution of IS*Aba*1 or IS*Aba*4 sequences upstream of various *bla*_OXA-23-_ and *bla*_OXA-51-like_ genes among the isolates that harbored ≥1 carbapenemase genes; and also their resistance rate against carbapenems. The presence of IS*Aba* upstream of the *bla*_OXA-51-like_ and*bla*_OXA-23-like_ genes was associated with high rate of carbapenem resistance. Among all *bla*_OXA-51-like^+^_ or *bla*_OXA-23-like^+^_ isolates, almost 32% (*n* = 27) lacked either IS*Aba*1, or IS*Aba*4 sequences. CR-AB isolates were consistently more resistant to doripenem than to imipenem, regardless of their *bla*_OXA-_ genes (Table 4). There was no marked difference in carbapenem resistance rates whether the isolates harbored the “*bla*_OXA-51-like_ gene alone,” or “*bla*_OXA-51-like_ plus *bla*_OXA-24-like_” genes. Overall, the IS*Aba*1 element was more often associated with *bla*_OXA-51-like_ gene (20–100%) than with *bla*_OXA-23-like_ genes; and IS*Aba*^+^ isolates showed high rates of carbapenem resistance, especially against doripenem. Despite this high resistance rate, 13% of *bla*_OXA-23-like^+^_/*bla*_OXA-51^+^_ isolates that harbored both IS*Aba*1 and IS*Aba*4 were imipenem susceptible (Table 4). Among IS*Aba*^+^ isolates, these elements were upstream of the *bla*_OXA-51-like_ gene in 60% (31/52) of the isolates, whereas only 31% harbored IS*Aba*1 upstream of the *bla*_OXA-23-like_ gene. All 13 (15%) CR-AB isolates with *bla*_OXA-51-like_ gene as their sole carbapenamase gene had IS*Aba*1 elements. Interestingly, even though test isolates showed an overall high resistance rate against carbapenems, 32% of isolates did not harbor either IS*Aba*1 or IS*Aba*4 elements. By and large, there was no marked change in resistance rate among isolates that harbored the *bla*_OXA-24-like_ gene in combination with other carbapenemase genes (Table 4).

**Table 4:** **Frequency of IS*Aba*1 or IS*Aba*4 sequences upstream of various *bla*_OXA-_ genes among test CR-AB isolates that harbored ≥ 1 carbapenemase genes, and the comparison of percent resistance against carbapenems among CR-AB isolates according to the isolate's *bla*_OXA-_ gene combination**.

**No**.	**Carbapenemase gene(s) of CR-AB isolates (Total = 85)**	**% of isolates with insertion sequences**					**% Carbapenem Resistance^a^**	
		**IS*Aba*1 on *bla*_OXA-51-like_(*n* = 31)**	**IS*Aba*1 on *bla*_OXA-23-like_(*n* = 16)**	**IS*Aba*1 on *bla*_OXA-51-like_and *bla*_OXA-23-like_ (*n* = 5)**	**IS*Aba*4 on *bla*_OXA-23-like_ (*n* = 6)**	**Without IS*Aba* (*n* = 27)**	**DOR^b^**	**IPM**
1	*bla*_OXA-51-like_ (*n* = 13; 15%)	38	–	–	–	62	77	46
2	*bla*_OXA-51-like_ and *bla*_OXA-23-like_ (*n* = 54; 63%)	37	30	9	9	15	100	72
3	*bla*_OXA-51-like_ and *bla*_OXA-24-like_(*n* = 10; 12%)	30	–	–	–	70	90	40
4	*bla*_OXA-51-like_, *bla*_OXA-23-like_ and *bla*_OXA-24-like_(*n* = 5; 6%)	20	0	0	20	60	100	80
5	*bla*_OXA-51-like_, *bla*_OXA-23-like_, *bla*_OXA-24-like_ and *bla*_OXA-58-like_ (*n* = 1; 1%)	100	0	0	0	0	100	100
6	*bla*_OXA-51-like_ and *vim*-2 (*n* = 2; 3%)	50	0	0	0	50	50	50

a *MIC ≤ 8 ug/ml*.

b *DOR, doripenem; IPM, imipenem*.

## Discussion

Infections caused by carbapenem-resistant *A. baumannii* are among the most difficult to treat, especially among ICU patients (Alfandari et al., 2014). In several countries, including Iran, clinicians face serious challenges in choosing an effective combination of antimicrobial agents while treating patients with severe nosocomial CR-AB infections. Efforts to control MDR-AB outbreaks have prompted widespread use of antimicrobials, such as tigecycline and colistin, as therapeutic measures to combat severe infections (Garnacho-Montero et al., 2015). However, appropriate treatment and effective infection control measures require local susceptibility patterns, as well as molecular epidemiologic data, such as the *bla*_OXA_ gene status of CR-AB isolates. Several surveillance studies have reported widespread nosocomial outbreaks of OXA-type producing *A. baumannii*, and a high prevalence of *bla*_OXA_ gene-carrying CR-AB in Iran, but data regarding their *bla*_OXA-23-like_ gene is not available (Moradi et al., 2015).

In the present study, we have genetically evaluated *bla*_OXA-23-like^+^_ CR-AB isolates and found high genotypic diversity among the isolates, including variants with new *bla*_OXA-23-like_ gene mutations. These mutations were associated with up to four-fold increases in MIC levels against imipenem, as compared to non-mutant isolates. Mutations in certain codons associated with a high degree of resistance to imipenem. For instance, substitutions at position 355 of the *bla*_OXA-23-like_ gene (i.e., the frame-shift mutation) were associated with high-level resistance, whereas position 256 mutations were associated with low-level resistance. Surprisingly, newly-found mutations correspond to regions of the carbapenemase molecule that are outside the standard “S-T-F-K, S-X-I, Y-G-N” and “K-S-G” oxacillinase motifs (Couture et al., 1992), suggesting that configurational changes due to novel mutations may also affect oxacillinase activity against carbapenems.

Although a high frequency of MDR (69%) and XDR (24%) *A. baumannii* isolates from this region is consistent with previous reports (D'Arezzo et al., 2011; Potron et al., 2011; Sung et al., 2011), our finding of 26 and 6% increases in resistance to tigecycline and colistin, respectively, (Bahador et al., 2014) is quite worrisome. Fortunately, while all isolates harboring mutations in *bla*_OXA-23-like_ genes showed resistance to carbapenem-class antibiotics; they were susceptible to tigecycline and/or tobramycin, which concurs with a recent report that shows potential activity of a number of combinations against MDR *A. baumannii* (Garnacho-Montero et al., 2015). Our data regarding to a high prevalence of *bla*_OXA-23-like_ genes among CR-AB from Tehran confirms previous reports (Shahcheraghi et al., 2011), and implies that extra efforts should be focused on controlling the spread of *bla*_OXA-23-like^+^_ *A. baumannii* in this area. Clonal outbreaks of OXA-23-producing CR-AB have been reported from several countries (Mugnier et al., 2010). Interestingly, isolates in this study did not harbor any NDM-1 metallo-β-lactamase genes, nor the “*bla*_*SPM*−1_ and *bla*_*GES*−1_” genes, which have recently been reported from India and Pakistan (Jones et al., 2014; Sartor et al., 2014), and Tehran (Shahcheraghi et al., 2011), respectively. However, we detected three mutant *bla*_OXA-23-like_ genes with identical sequences reported from China.

The data on the presence of IS*Aba*1 sequences upstream from *bla*_OXA_ genes and enhancement of OXA–enzyme expression confirms previous reports (Sung et al., 2011); however, these findings are in contrast with a report from northwestern Iran, which detected no IS*Aba*1 sequences upstream of the *bla*_OXA_ gene (Peymani et al., 2012). Together, these results suggest that the CR-AB populations in various parts of Iran are diverse and distinct, which may hint on probable differences in antimicrobial management of *A. baumannii* infections in various regions. Moreover, most of the *bla*_OXA-23_ mutant *A. baumannii* isolates were obtained from urine and sputum samples, suggesting that specific infection control protocols regarding urinary catheters and ventilators are possible primary sources of CR-AB transmission.

Although increased imipenem resistance due to a mutation in the *bla*_OXA-23_ gene has been reported previously (Lin et al., 2011), to the best of our knowledge, this is the first report of CR-AB *bla*_OXA-23_ gene mutants from Iran. It is noteworthy that the TUMS/BTRF661 strain showed the highest MIC (128 μg/mL), implying a greater influence of the frame-shift mutation on carbapenem resistance than any of the substitution mutations. Our future studies will focus on exploring the difference in the MICs of the various mutants (16 vs. 128 μg/mL) and the potential complex interactions between antimicrobial agents and carbapenemase at the molecular level, where the position of the affected motif plays a critical role. While production of carbapenemase remains to be the chief mechanism of carbapenem-resistance in *A. baumannii*, whether additional factors, such as alterations in outer membrane permeability, efflux pumps as with AdeABC (Potron et al., 2015), or OprD porin (Potron et al., 2015), contribute to carbapenem-resistance amongmutants. The variability in the β-lactamase activity and MIC values of variants that share a mutation, suggests that other factors play a role in high MIC levels among mutant CR-AB isolates, and they remain to be explored.

Assuming future confirmation of the correlation of specific mutations with high MICs, carbapenem resistance levels may be predictable by DNA sequence-based detection methods. Also, determination of predominant *bla*_OXA-23_ genotype(s) of isolates in various areas and their *bla*_OXA-23-like_ gene mutations may serve as a tool for molecular epidemiologic investigations to control the spread of CR-AB infections. While the present study focused on the chromosomal OXA-encoding genes in CR-AB, we also plan to explore the role of mutations in plasmid-encoded *bla*_OXA-23-like_ genes among carbapenem-resistant *A. baumannii*, since many of these genes are plasmid-borne.

In conclusion, we report the identification of CR-AB variants that harbor *bla*_OXA-23-like_ gene mutations, which are associated with an increased MIC against imipenem. Several *bla*_OXA-23-like_ mutant isolates are widespread and have been reported from the USA, and China. The detection of new *bla*_OXA-23_ mutant isolates from Iran highlights the importance of concerted efforts, at the national and global levels, toward the control of carbapenem-resistance among *A. baumannii* isolates worldwide.

## Funding

This research has been supported by Tehran University of Medical Sciences and Health Services grant no. 89. 01-30-10430.

## Conflict of interest statement

The authors declare that the research was conducted in the absence of any commercial or financial relationships that could be construed as a potential conflict of interest.

## Supplementary material

Supplemental Figure 1 **Presentation of study strategy to select *bla*_OXA-23^+^_*A. baumannii* clinical isolates for AFLP genotype determination and DNA sequence analysis**. Step-wise selection of blaOXA^+^ isolates was carried out using 2 sets of multiplex PCR assay, followed by AFLP genotypic analysis and DNA sequencing of *bla*_OXA-23-like_ gene amplicons.

Supplemental Figure 2 **Agarose gel electrophoresis analysis of PCR amplicons specific for Ambler Class A and B carbapenemases**. Genomic DNA from clinical *A. baumannii* isolates were analyzed by uniplex and multiplex PCR assay as described (M&M). Lanes 1 through 6; specific bands for (1) NDM1, (2) IMP1, (3) VIM2, (4) SPM1, (5) *bla*_*GES*_, and (6) *KPC* encoding genes. Lane 7; AB-hexaplex (AB-h) PCR products of the above genes. Lane 8; 100 bp DNA markers.

Supplemental Figure 3 **Comparison between DNA sequences of AB0057Ref.seq and PCR amplicon sequences obtained from mutant CR-AB isolates in genotype groups with >2 members showing a 10-nucleotide span in each direction of the point of mutation (total 21 nucleotides). (A)** Alignment of amplicons from three isolates of AFLP group L (i.e., TUMS/BTRF443, L2, and L3) as compared to the AB0057Ref.seq sequence, with the mutation at position 766. **(B)** Alignment of AFLP groups E, C, and K amplicons with the AB0057Ref.seq reference sequence, with the mutation at position 771. In both panels (^*^) indicates sequence identity (or homology), and (−) shows the position of carbapenemase gene mutation.
