# Erratum: Genotypic and Antimicrobial Susceptibility of Carbapenem-Resistant *Acinetobacter baumannii*: Analysis of IS*Aba* Elements and *bla*_OXA-23-like_ Genes Including a New Variant

**DOI:** 10.3389/fmicb.2015.01430

**Published:** 2015-12-14

**Authors:** Frontiers Production Office

**Affiliations:** Frontiers Production Office, FrontiersSwitzerland

**Keywords:** *Acinetobacter baumannii*, *bla*_OXA-23-like_ gene, carbapenemase, novel mutations

Reason for Erratum:

Due to a typesetting error in the title, the IS*Aba* Elements were reported incorrectly as “is *Aba*.” The correct title is: Genotypic and Antimicrobial Susceptibility of Carbapenem-Resistant *Acinetobacter baumannii*: Analysis of IS*Aba* Elements and *bla*_OXA-23-like_ Genes Including a New Variant.

The publisher apologizes for this error and the original article has been updated. This error does not change the scientific conclusions of the article in any way.

