# Supplementary figures and images for: Genotypic and Antimicrobial Susceptibility of Carbapenem-resistant Acinetobacter baumannii: Analysis of ISAba Elements and blaOXA-23-like Genes Including a New Variant
Source: Front Microbiol. 2015 Nov 13;6:1249. doi: 10.3389/fmicb.2015.01249 (PMC4643144; doi:10.3389/fmicb.2015.01249)

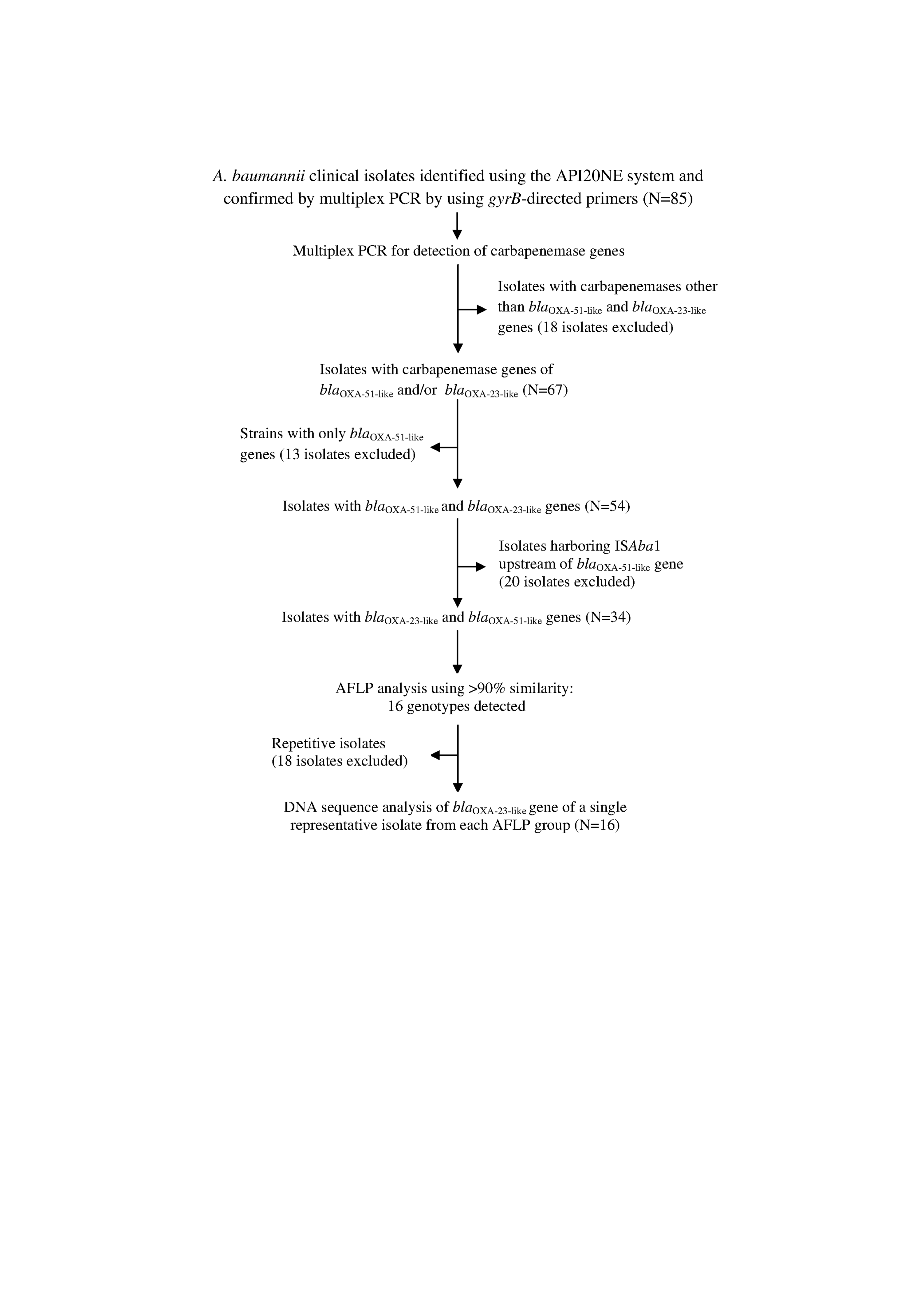

Supplement: Supplemental Figure 1 — Presentation of study strategy to select blaOXA-23+A. baumannii clinical isolates for AFLP genotype determination and DNA sequence analysis. Step-wise selection of blaOXA+ isolates was carried out using 2 sets of multiplex PCR assay, followed by AFLP genotypic analysis and DNA sequencing of blaOXA-23-like gene amplicons. [file Image1.TIFF]

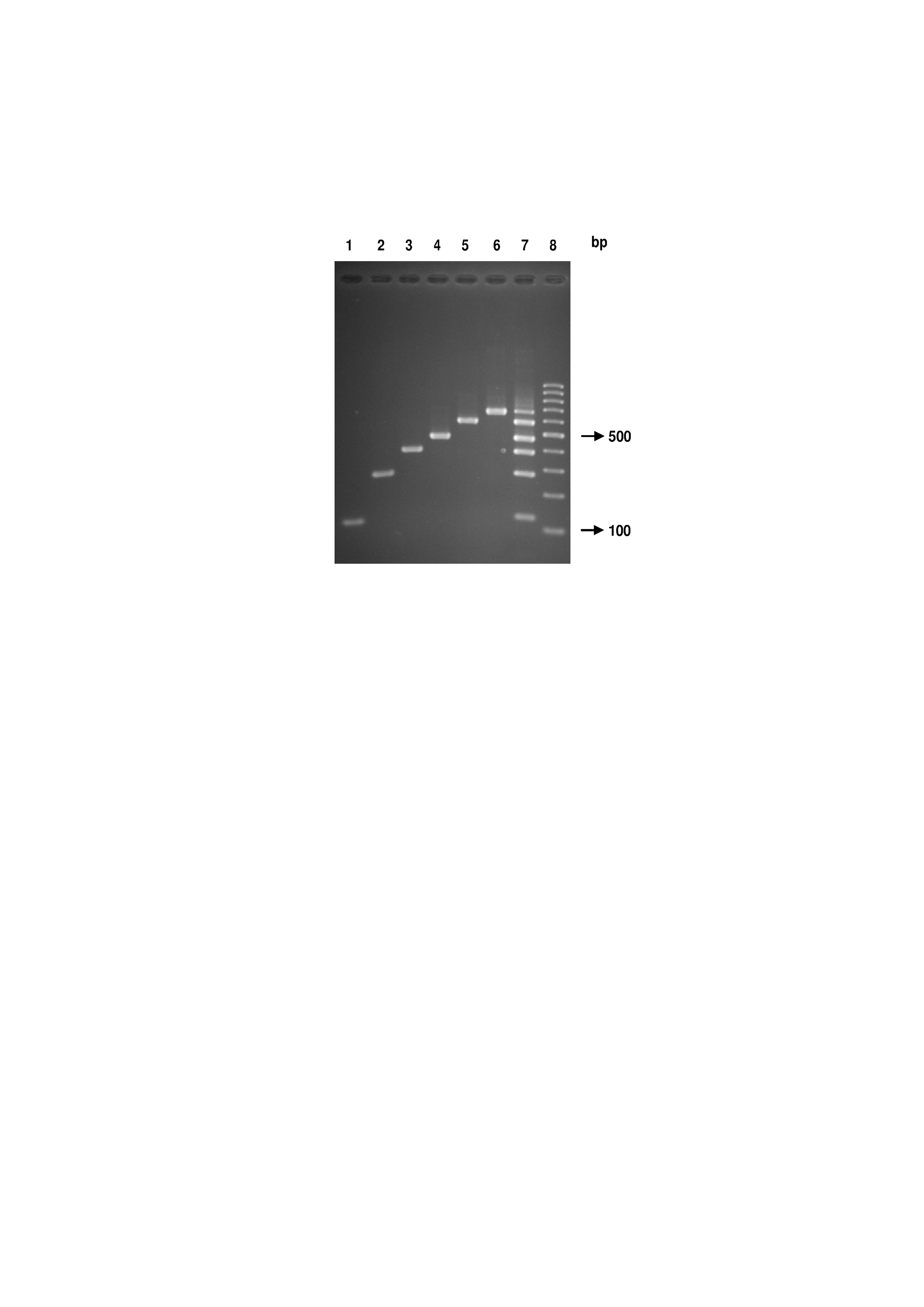

Supplement: Supplemental Figure 2 — Agarose gel electrophoresis analysis of PCR amplicons specific for Ambler Class A and B carbapenemases. Genomic DNA from clinical A. baumannii isolates were analyzed by uniplex and multiplex PCR assay as described (M&M). Lanes 1 through 6; specific bands for (1) NDM1, (2) IMP1, (3) VIM2, (4) SPM1, (5) blaGES, and (6) KPC encoding genes. Lane 7; AB-hexaplex (AB-h) PCR products of the above genes. Lane 8; 100 bp DNA markers. [file Image2.TIFF]

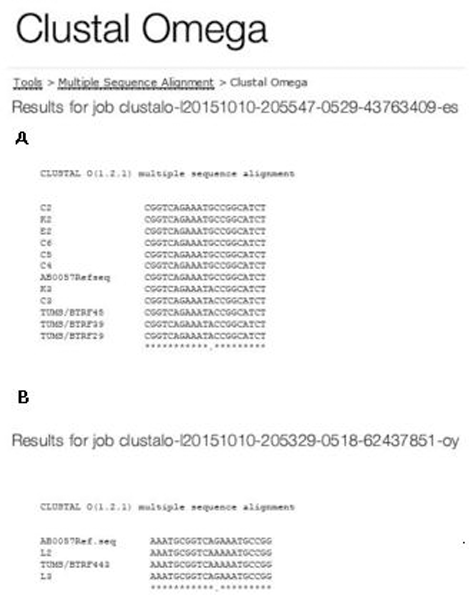

Supplement: Supplemental Figure 3 — Comparison between DNA sequences of AB0057Ref.seq and PCR amplicon sequences obtained from mutant CR-AB isolates in genotype groups with >2 members showing a 10-nucleotide span in each direction of the point of mutation (total 21 nucleotides). (A) Alignment of amplicons from three isolates of AFLP group L (i.e., TUMS/BTRF443, L2, and L3) as compared to the AB0057Ref.seq sequence, with the mutation at position 766. (B) Alignment of AFLP groups E, C, and K amplicons with the AB0057Ref.seq reference sequence, with the mutation at position 771. In both panels (*) indicates sequence identity (or homology), and (−) shows the position of carbapenemase gene mutation. [file Image3.TIFF]
